# Impact of the LDL subfraction phenotype on Lp-PLA2 distribution, LDL modification and HDL composition in type 2 diabetes

**DOI:** 10.1186/1475-2840-12-112

**Published:** 2013-08-05

**Authors:** Jose Luis Sánchez-Quesada, Irene Vinagre, Elena De Juan-Franco, Juan Sánchez-Hernández, Rosa Bonet-Marques, Francisco Blanco-Vaca, Jordi Ordóñez-Llanos, Antonio Pérez

**Affiliations:** 1 Biomedical Research Institute IIB Sant Pau, Cardiovascular Biochemistry Group, C/ Antoni Maria Claret, 167, 08025 Barcelona, Spain; 2 Endocrinology and Nutrition Department, Hospital de la Santa Creu i Sant Pau, C/ Mas Casanovas, 90, 08041 Barcelona, Spain; 3 CIBER for the Study of Diabetes and Associated Metabolic Diseases (CIBERDEM), Barcelona, Spain; 4 Metabolic Factors of Cardiovascular Risk Group, Biomedical Research Institute IIB Sant Pau, Barcelona, Spain; 5 Biochemistry and Molecular Biology Department, Universitat Autònoma de Barcelona, Cerdanyola del Vallés, Spain

**Keywords:** Low-density lipoprotein, High-density lipoprotein, Lipoprotein-associated phospholipase A2, Modified LDL, Type 2 diabetes, LDL subfraction phenotype

## Abstract

**Background:**

Qualitative alterations of lipoproteins underlie the high incidence of atherosclerosis in diabetes. The objective of this study was to assess the impact of low-density lipoprotein (LDL) subfraction phenotype on the qualitative characteristics of LDL and high-density lipoprotein (HDL) in patients with type 2 diabetes.

**Methods:**

One hundred twenty two patients with type 2 diabetes in poor glycemic control and 54 healthy subjects were included in the study. Patients were classified according to their LDL subfraction phenotype. Seventy-seven patients presented phenotype A whereas 45 had phenotype B. All control subjects showed phenotype A. Several forms of modified LDL, HDL composition and the activity and distribution of lipoprotein-associated phospholipase A2 (Lp-PLA2) were analyzed.

**Results:**

Oxidized LDL, glycated LDL and electronegative LDL were increased in both groups of patients compared with the control group. Patients with phenotype B had increased oxidized LDL and glycated LDL concentration than patients with phenotype A. HDL composition was abnormal in patients with diabetes, being these abnormalities more marked in patients with phenotype B. Total Lp-PLA2 activity was higher in phenotype B than in phenotype A or in control subjects. The distribution of Lp-PLA2 between HDL and apoB-containing lipoproteins differed in patients with phenotype A and phenotype B, with higher activity associated to apoB-containing lipoproteins in the latter.

**Conclusions:**

The presence of LDL subfraction phenotype B is associated with increased oxidized LDL, glycated LDL and Lp-PLA2 activity associated to apoB-containing lipoproteins, as well as with abnormal HDL composition.

## Background

Atherosclerosis-related pathologies are the main cause of mortality and morbidity in type 2 diabetes. Plasma lipid profile, including hypertriglyceridemia, low high-density lipoprotein (HDL) cholesterol levels and hyperapolipoprotein B, is frequently abnormal in these subjects and is a cause of the high prevalence of macrovascular and microvascular complications [1,2]. However, these quantitative abnormalities per se cannot explain all the increase in cardiovascular risk (CVR) in diabetes, and it is currently accepted that alterations in the qualitative properties of low-density lipoprotein (LDL) and HDL underlie the development of atherosclerosis [1]. The concurrence of hypertriglyceridemia and low HDL cholesterol usually promote the formation of small dense LDL (sdLDL) particles. This set of features, known as diabetic dyslipidemia, is partly for environmental reasons but also has a significant genetic component [3]. The predominance of sdLDL is known as LDL subfraction phenotype B, in contrast to phenotype A, which is characterized by large buoyant LDL particles. Phenotype A is predominant in normolipemic subjects with low CVR, whereas phenotype B is associated with pathological situations with increased CVR, including type 2 diabetes [4].

Other pathological features, such as hyperglycemia, lipoperoxidation and systemic inflammation are exacerbated in diabetes. These processes alter the lipoprotein function [5], favoring the formation of modified forms of LDL, such as glycosylated LDL (glLDL), oxidized LDL (oxLDL) and electronegative LDL (LDL(−)). A common feature shared by glLDL and oxLDL is the increased negative electric charge. LDL(−), therefore, includes oxLDL and glLDL, but a major fraction of LDL(−) particles is generated by alternative mechanisms, such as lipolysis and non-esterified fatty acid (NEFA)-loading [6]. This heterogeneity adds value to the measurement of LDL(−), which can be considered as a pool of modified LDL particles in plasma. The proportion of oxLDL, glLDL and LDL(−) is increased in subjects with high CVR, including diabetes [7]. These modified LDLs have a high content of lipid metabolites with high inflammatory potential, including lysophospholipids, whose concentration is increased in diabetes [8].

Diabetes also alters normal HDL function. The antiatherogenic properties of HDL, including its key role in reverse cholesterol transport and its antioxidant properties, are disturbed in these patients [9]. Both functions are modulated by changes in the relative composition of lipids and proteins in HDL, and also by enzymatic activities associated to HDL, such as paraoxonase 1 (PON1) and lipoprotein-associated phospholipase A2 ((Lp-PLA2), also known as platelet-activating factor acetylhydrolase). PON1 is exclusively bound to HDL and it has been suggested that it has an antioxidant function by deactivating lipid peroxides [10]. In contrast to PON1, Lp-PLA2 can associate with HDL, LDL and VLDL. Controversy exists as to whether Lp-PLA2 has a pro- or anti-inflammatory action. It has been recently suggested that the relative distribution of Lp-PLA2 between LDL and HDL determines its pro- or anti-inflammatory action. According to this assumption, Lp-PLA2 in HDL is anti-inflammatory whereas Lp-PLA2 associated to apoB-containing lipoproteins is pro-inflammatory [11]. For this reason, the relative distribution of Lp-PLA2, rather than its total activity, could be a better CVR marker.

In vitro studies have shown that sdLDL is prone to modification and it is also known that HDL from patients with type 2 diabetes has an impaired function. However, to our knowledge, no previous study has compared diabetic patients with phenotype A and phenotype B. This would allow specifically assessing the impact of LDL subfraction phenotype on the distribution of Lp-PLA2 and other qualitative characteristics of HDL and LDL. The aim of the current work was to quantify modified forms of LDL, to study the composition of HDL and to determine the distribution of Lp-PLA2 in patients with poorly-controlled type 2 diabetes who were classified according to their LDL subfraction phenotype.

## Methods

### Study population

One hundred twenty two consecutive poor controlled type 2 diabetes subjects who attended the outpatient diabetic clinic during 2007–2008 to optimize glycemic control were recruited. Previous hypoglycemic treatment consisted of diet (22%), oral agents (21.1%), insulin plus oral agents (29.4%) and insulin (27.5%). Regarding hypolipidemic drugs, 25.4% of patients were treated with statins or statin plus ezetimibe, 4.1% with fibrate and 2.5% with statin plus fibrate. Patients with acute or chronic infections, active inflammatory diseases or treatment with anti-inflammatory drugs were excluded. High sensibility C-reactive protein (hsCRP, Roche Diagnostics) was routinely measured and subjects with values higher than 20 mg/L were excluded from the study. Hypertension was defined as subjects with a systolic blood pressure of ≥140 mm Hg, with a diastolic blood pressure of ≥90 mm Hg, or those who were receiving antihypertensive therapy at the time of examination. Coronary Heart Disease (CHD) was defined as documented diagnosis of CHD, self-reported positive history of CHD or electrocardiogram (ECG) positive for CHD. Fifty four normoglycemic and normolipemic healthy subjects were included as control group. The study was conducted in accordance with the principles of the Declaration of Helsinki and was approved by the Hospital Ethics Committee. All patients and control subjects gave informed consent.

### Laboratory determinations

Lipid profile, modified LDLs, HDL composition and Lp-PLA2 activity distribution were determined from plasma obtained in EDTA-containing Vacutainer tubes. PON1 activity was determined from sera obtained in non-additive Vacutainer tubes. HbA1c was measured by ion-exchange high-performance liquid chromatography (HPLC; Variant, Bio-Rad).

Lipid profile included total cholesterol (Roche) and triglyceride (Roche), very-low density lipoprotein (VLDL), LDL and HDL cholesterol, total non-esterified fatty acids (NEFA) (Wako), apoB (Roche), apoA-I (Roche) and apoA-II (Kamiya Biomedical). Cholesterol of lipoprotein fractions was measured using a direct method to quantify HDL cholesterol (HDL-C plus, Roche) or by ultracentrifugation when the triglyceride concentration was higher than 3 mmol/L, according to NCEP recommendations [12]. All determinations were performed in a Hitachi 917 autoanalyzer.

LDL size was determined by non-denaturing polyacrylamide gradient (2–16%) gel electrophoresis (GGE), as described [13]. LDL subfraction phenotype B was defined by a predominant LDL diameter lower than 25.5 nm, whereas phenotype A subjects had a LDL diameter higher than 25.5 nm.

oxLDL (Mercodia) and glLDL (Exocell/Glycacor) were quantified by commercial ELISA. For LDL(−) quantification, total LDL was previously isolated by sequential ultracentrifugation (density range 1.019-1.050 g/mL). LDL(−) proportion was determined from total LDL by anion-exchange chromatography in a MonoQ HR 5/5 column (GE Healthcare) using a NaCl stepwise gradient [14].

HDL was isolated by sequential ultracentrifugation (density range 1.063-1.210 g/mL), using KBr gradients. All steps were performed at 4°C and all KBr solutions contained 1 mmol/L EDTA and 2 μmol/L BHT to prevent lipoperoxidation during isolation procedures. HDL composition was determined by measuring the content of cholesterol (Roche), triglyceride (Roche), phospholipid (Wako), NEFA (Wako), apoA-I (Roche) and apoA-II (Kamiya), in a Hitachi 917 autoanalyzer.

PON-1 activity in serum was measured using phenylacetate as substrate, as described [15]. Lp-PLA2 activity was measured using 2-thio-PAF (Cayman) as substrate, according to the manufacturer’s instructions [16]. To determine the distribution of Lp-PLA2 between lipoprotein fractions, apoB-containing lipoproteins were precipitated from serum using dextran sulfate, as described [17]. Briefly, 200 μL of serum were mixed with 50 μL of dextran sulfate reagent, incubated for 5 min at room temperature, and centrifuged for 10 min at 10,000 g. The supernatant (depleted of apoB-containing lipoproteins) was collected and assayed for HDL-associated Lp-PLA2 activity.

### Statistical analysis

Statistical analysis was performed using SPSS 19 (SPSS Inc). Before statistical analysis, normal distribution and homogeneity of the variances were tested using Kolmogorov-Smirnov and Levène tests, respectively. Data that were not normally distributed were logarithmically transformed before analysis. Analysis of variables was performed by one-way analysis of variance (ANOVA). ANCOVA test was used to assess if the differences seen in ANOVA persisted after adjustment for triglycerides and HbA1c. Differences between two groups were compared using the Student’s T test for unpaired data (variables with normal distribution) or the Mann–Whitney U test (variables with non-normal distribution). Correlations between parameters were analyzed using the Pearson R test for variables with normal distribution and the Spearman test for variables with non-normal distribution. Data are expressed as mean ± SD. P < 0.05 was considered significant.

## Results

Table 1 shows anthropometrics, clinical characteristics and lipid profile of patients and controls. One third (n = 45) of patients with type 2 diabetes presented phenotype B (LDL size < 25.5 nm), in contrast to the control group in which all subjects (n = 54) had phenotype A. Table 1 shows the lipid profile of patients with type 2 diabetes and control subjects. Both groups of patients had higher plasma levels of triglyceride, VLDL cholesterol, apoB and NEFA and lower concentration of HDL cholesterol and apoA-I than control subjects. The lipid profile was worse in patients with phenotype B. The differences observed in the two groups of patients compared to controls were larger in phenotype B patients, who also presented higher total cholesterol and LDL cholesterol than controls. The differences between phenotype A and phenotype B patients included higher total cholesterol, triglyceride, VLDLc, LDLc, apoB and lower HDLc and apoA-I in patients with phenotype B than in patients with phenotype A (Table 1).

**Table 1:** Anthropometrics, clinical characteristics and lipid profile of patients with type 2 diabetes and control subjects

	**Type 2 diabetes phenotype A (n = 77)**	**Type 2 diabetes phenotype B (n = 45)**	**Control group**	**ANOVA**
			**(n = 54)**	**p-value**
Age (years)	60 ± 11	56 ± 11 ^a^	56 ± 14 ^a^	0.108
Male (%)	65.9	64.6	57.4 ^a, b^	<0.001
BMI (kg/m2)	29.6 ± 6.2	31.1 ± 4 ^a^	25.9 ± 3.6 ^a, b^	<0.001
Waist (cm)	104 ± 15	108 ± 13 ^a^	92 ± 11 ^a, b^	<0.001
Diabetes duration (years)	12 ± 11	9 ± 8 ^a^	-	
Insulin use (%)	58	52 ^a^	-	
Lipid lowering drugs (%)	30	28	-	
HbA1c (%)	9.1 ± 2.1	9.6 ± 2.3 ^a^	5.3 ± 0.6 ^a, b^	<0.001
Hypertension (%) ^1^	69.4	68.2	-	
Retinopathy (%)	29.6	29.5	-	
Albuminuria (%)	24	29.6 ^a^	-	
Coronary heart disease (%) ^2^	5.6	15.9 ^a^	-	
Total cholesterol (mmol/L)	4.78 ± 1.09	5.64 ± 1.53 ^a^	5.00 ± 0.79 ^b^	0.004
Triglycerides (mmol/L)	1.46 ± 0.79	3.89 ± 3.30 ^a^	0.87 ± 0.37 ^a,b^	<0.001
VLDLc (mmol/L)	0.65 ± 0.30	1.43 ± 1.12 ^a^	0.40 ± 0.17 ^a,b^	<0.001
LDLc (mmol/L)	2.86 ± 0.95	3.10 ± 0.96 ^a^	2.94 ± 0.66 ^b^	0.281
HDLc (mmol/L)	1.28 ± 0.33	1.02 ± 0.27 ^a^	1.64 ± 0.37 ^a,b^	<0.001
NEFA (mmol/L)	0.64 ± 0.29	0.90 ± 0.48	0.41 ± 0.19 ^a,b^	<0.001
apoB (g/L)	0.93 ± 0.32	1.09 ± 0.28 ^a^	0.80 ± 0.19 ^a,b^	<0.001
apoA-I (g/L)	1.46 ± 0.24	1.37 ± 0.25 ^a^	1.57 ± 0.31 ^a,b^	0.001
apoA-II (g/L)	0.33 ± 0.07	0.34 ± 0.06	0.35 ± 0.05	0.081
LDL size (nm)	26.0 ± 0.4	25.0 ± 0.4 ^a^	26.3 ± 0.1 ^b^	<0.001

^a^, P < 0.05 vs phenotype A patients; ^b^, P < 0.05 vs phenotype B patients. ^1^ Hypertension was defined as a systolic blood pressure of ≥140 mm Hg, a diastolic blood pressure of ≥90 mm Hg or those who were receiving antihypertensive therapy at the time of examination. ^2^ Coronary Heart Disease (CHD) was defined as documented diagnosis of CHD, self-reported positive history of CHD or ECG positive for CHD.

Regarding modified LDLs, both groups of patients had higher plasma concentration of oxLDL, glLDL and LDL(−) than control subjects (Table 2). oxLDL and glLDL concentrations were higher in patients with phenotype B than in patients with phenotype A. In contrast, the proportion of LDL(−) was similar between both groups of patients.

**Table 2:** Modified LDLs, HDL composition and Lp-PLA2 activity in patients with type 2 diabetes and control subjects

	**Type 2 diabetes phenotype A (n = 77)**	**Type 2 diabetes phenotype B (n = 45)**	**Control group**	**ANOVA**
			**(n = 54)**	**p-value**
Modified LDL				
Oxidized LDL (U/L)	61.4 ± 20.0	78.6 ± 26.7 ^a^	51.2 ± 19.3 ^a,b^	0.005
Glycated LDL (mg/dL)	2.17 ± 0.94	2.77 ± 1.41 ^a^	1.85 ± 0.73 ^a,b^	0.030
LDL(−) (%)	7.6 ± 3.5	7.7 ± 3.1	6.1 ± 2.0 ^a,b^	0.017
HDL composition				
Cholesterol (%)^1^	17.1 ± 2.3	15.6 ± 3.0 ^a^	17.3 ± 2.3 ^b^	0.204
Trigycerides (%)	4.3 ± 1.7	6.7 ± 1.9 ^a^	3.1 ± 1.0 ^a,b^	<0.001
Phospholipids (%)	33.5 ± 4.3	31.3 ± 3.3 ^a^	32.8 ± 3.7	0.076
apoA-I (%)	31.9 ± 5.8	32.8 ± 6.3	34.3 ± 5.7 ^a,b^	0.193
apoA-II (%)	13.2 ± 3.4	13.6 ± 2.6	12.5 ± 2.9 ^a,b^	0.265
NEFA(mol/mol apoA-I)	1.1 ± 0.6	1.0 ± 0.6	1.0 ± 0.9	0.907
Ratio lipid/protein	1.20 ± 0.28	1.19 ± 0.27	1.16 ± 0.27 ^a,b^	0.539
Ratio A-I/A-II	2.61 ± 0.92	2.54 ± 0.81	2.98 ± 1.12 ^a,b^	0.112
Ratio chol/trigl	4.65 ± 2.19	2.57 ± 0.91	6.32 ± 2.68 ^a,b^	<0.001
PON1 activity	78.5 ± 25.5	82.6 ± 25.5	82.5 ± 23.1	0.152
(μmol/min/mL)				
Lp-PLA2 activity(μmol/min/mL)				
Total Lp-PLA2	20.1 ± 6.6	23.0 ± 7.2 ^a^	19.1 ± 6.2 ^b^	0.627
HDL-Lp-PLA2	6.8 ± 2.7	7.1 ± 3.5	6.2 ± 2.6	0.995
apoB-associated-Lp-PLA2	13.3 ± 4.8	15.9 ± 4.6 ^a^	12.9 ± 3.3 ^b^	0.539
% HDL-Lp-PLA2	33.9 ± 10.5	30.9 ± 11.4 ^a^	32.4 ± 10.6	0.510
% apoB-associated-Lp-PLA2	66.1 ± 19.4	69.1 ± 21.2 ^a^	67.6 ± 19.8	0.510

^a^, P < 0.05 vs phenotype A patients; ^b^, P < 0.05 vs phenotype B patients.

^1^ Composition is expressed as the percentage of the mass of cholesterol, triglycerides, phospholipids, apoA-I and apoA-II.

The composition analysis of isolated HDL particles showed that both groups of patients had an abnormal composition compared to control samples (Table 2), with some of these alterations being stronger in patients with phenotype B. Differences between patients with phenotype B and controls included a lower content of cholesterol and apoA-I, and a higher content of triglycerides and apoA-II. Similar differences were observed when controls were compared with patients with phenotype A, except that cholesterol content was similar in both groups. As a consequence, the apoA-I/apoA-II ratio was lower and the lipid/protein ratio was higher in HDL from both groups of patients than in control subjects. Both ratios were similar in patients with phenotype A and phenotype B. These observations suggest that HDL in both groups of patients with diabetes has lower density and larger size than HDL in control subjects. Regarding PON1 activity, no difference was observed.

**Figure 1:**
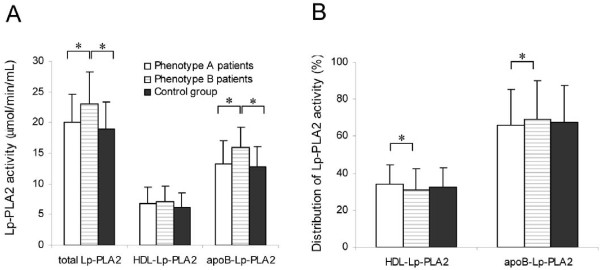
**Lp-PLA2 activity.** Enzymatic activity **(A)** and relative distribution between HDL and apoB-containing lipoproteins **(B)** of Lp-PLA2 in control subjects and patients with type 2 diabetes classified according their LDL subfraction phenotype. * indicates P < 0.05.

Total and apoB-associated Lp-PLA2 activity was higher in patients with phenotype B than in control subjects and in patients with phenotype A, and was similar in the two latter groups (Table 2, Figure 1A). No statistical difference was observed in the HDL-associated Lp-PLA2 activity. Consequently, the distribution of Lp-PLA2 between HDL and apoB-containing lipoproteins (Table 2, Figure 1B) was different between patients with phenotype A and those with phenotype B. The latter had lower proportion of Lp-PLA2 in HDL and higher proportion in apoB-containing lipoproteins than in patients with phenotype A.

Covariance analysis (ANCOVA) showed that the differences seen in apoB and glLDL disappeared after adjusting for TG. Differences observed in LDL(−) and oxLDL lost their significance after adjusting for HbA1c and TG (data not shown). These associations are expected and underline the known relation of LDL(−) and oxLDL with the presence of diabetes and hypertriglyceridemia, and of apoB and glLDL with hypertriglyceridemia. Table 3 shows the correlation analysis of LDL size with lipid profile, modified LDLs, HDL composition and Lp-PLA2 activity. In diabetic patients, significant correlations with the parameters of lipid profile were similar to those previously reported: LDL size correlated negatively with triglycerides, cholesterol, VLDLc, apoB and NEFA, and positively with HDLc and apoA-I. LDL size also correlated negatively with oxLDL and glLDL. Regarding HDL composition, LDL size correlated positively with the content of phospholipids, cholesterol and Lp-PLA2, and negatively with the content of triglycerides and apoA-I. The correlation analysis of the control group showed some differences compared to diabetic patients. Most of correlations observed between LDL size and lipid profile were similar to that in DM2 patients, except the positive correlation with HDLc and the negative correlation with plasma NEFA, which were lost in the control group. The negative correlation with glLDL as well as the correlations with some HDL components also were lost in control subjects. These observations suggest that the influence of LDL size on HDL composition and glLDL is stronger in diabetic patients than in control subjects.

**Table 3:** Correlation analysis of LDL size with lipid profile, modified LDLs, HDL composition and Lp-PLA2 activity in patients with type 2 diabetes and control subjects

	**DM2 patients**		**Control subjects**	
	R	P	R	P
HbA1c	−0.160	0.071	−0.103	0.601
Insulin	−0.076	0.418	0.008	0.937
Total cholesterol	−0.185	0.035	−0.326	0.016
Triglycerides	−0.553	< 0.001	−0.427	0.001
VLDLc	−0.513	< 0.001	−0.423	0.001
LDLc	−0.152	0.083	−0.333	0.014
HDLc	0.407	< 0.001	0.096	**0.491**
NEFA	−0.226	0.004	−0.042	**0.765**
apoB	−0.246	0.005	−0.458	< 0.001
apoA-I	0.264	0.002	−0.033	**0.811**
apoA-II	−0.068	0.472	−0.099	0.476
Oxidized LDL	−0.390	< 0.001	−0.472	< 0.001
Glycated LDL	−0.273	0.003	−0.151	**0.307**
LDL(-)	−0.020	0.828	0.030	0.849
Cholesterol in HDL	0.321	0.002	0.208	**0.244**
Trigycerides in HDL	−0.398	< 0.001	−0.150	**0.405**
Phospholipids in HDL	0.437	< 0.001	0.067	**0.712**
apoA-I in HDL	−0.205	0.047	0.086	0.635
apoA-II in HDL	−0.169	0.103	−0.069	0.739
NEFA in HDL	−0.010	0.925	0.086	0.635
PON1	0.016	0.867	0.024	0.872
Total Lp-PLA2 activity	−0.123	0.172	0.025	0.862
HDL-Lp-PLA2 activity	0.030	0.745	0.061	0.680
% of HDL-Lp-PLA2	0.191	0.034	0.064	0.662

Bold type indicates the loss of statistical significance in the control group compared to diabetic patients.

## Discussion

Several authors have reported differences in the qualitative characteristics of HDL and LDL between diabetic and healthy subjects. However, most of these studies analyzed separately LDL and HDL and, despite the high prevalence of LDL phenotype B in type 2 diabetes, these characteristics have not been usually compared classifying diabetic subjects according to their LDL subfraction phenotype. Regarding Lp-PLA2, its distribution has been analyzed comparing LDL phenotypes only in one study [18], but not in patients with diabetes. The novelty of our study is to analyze simultaneously oxLDL, glLDL, LDL(−), HDL composition and distribution of Lp-PLA2 in patients with type 2 diabetes classified according to LDL phenotype.

Results in the current study concur with previous findings reporting that oxLDL, glLDL and LDL(−) are increased in diabetes [7,19,20]. Our data show that oxLDL and glLDL are increased in patients with phenotype A compared to control subjects, but this situation is more marked in patients with phenotype B. The high concentration of oxLDL and glLDL observed in patients with phenotype B could be attributed to several mechanisms. It is known that sdLDL has an impaired plasma clearance, which could lead to increased residence time in blood and favor further modifications. This could be related to the high susceptibility to oxidation and to non-enzymatic glycosylation that has been demonstrated both “in vitro” and “in vivo” in sdLDL particles [5]. In this context, it is worthy mentioning the work by Younis et al., who recently reported that sdLDL concentration is an even stronger determinant of LDL glycation than hyperglycemia [21]. However, differences in oxLDL and glLDL between patients with phenotype A and control subjects still remained significant. Therefore, factors other than LDL size could account for these differences. First, hyperglycemia and increased oxidative stress, which are hallmarks of diabetes, affect circulating LDL, even in patients with phenotype A. Another possible explanation is related to the altered composition of HDL observed in both groups of patients with type 2 diabetes. Our results indicate that HDL composition was abnormal in both groups of patients, although alterations were stronger in phenotype B patients. These differences included apoA-I/apoA-II and lipid/protein ratios. A decrease of apoA-I in HDL with a concomitant increase of apoA-II produces particles with lower antioxidant capacity [15]. Likewise, higher lipid/protein ratio suggests an impairment of the antioxidant properties of HDL, since the HDL3 subfraction (with lower lipid/protein ratio) has a stronger antioxidant capacity than the HDL2 subfraction (with higher lipid/protein ratio) [22].

The alterations in the composition of HDL from patients with diabetes also point to a decreased ability to promote reverse cholesterol transport. An increased triglyceride and decreased cholesterol content in HDL has been related with impaired cholesterol efflux from adipose and hepatic cells [23]. This alteration was present in HDL isolated from our patients and could be indicative of such impairment. This would be pronounced in diabetic subjects with phenotype B in which the ratio cholesterol/triglyceride in HDL is 2.5-fold lower than in control subjects.

Although the concentration of oxLDL and glLDL was increased in patients with phenotype B, the relative content of LDL(−) was not affected by the presence of sdLDL particles, because there was no difference between phenotype A and phenotype B patients. This observation suggests that although oxidation and glycosylation could be partially involved in the generation of LDL(−), these would not be the major mechanisms in diabetes. Other mechanism involved in the formation of LDL(−) in diabetes is NEFA-loading. [14]. As a consequence, the increased plasma level of NEFA is probably a major cause of increased LDL(−) in diabetes.

The role of Lp-PLA2 in atherogenesis is controversial. Lp-PLA2 is thought to play a role in the prevention of oxidative modifications [24], but it has been positively associated to coronary heart disease. Most epidemiological studies have found a relationship between Lp-PLA2 and coronary events or ischemic stroke [25]. However, Lp-PLA2 shows a strong association with total and LDL cholesterol levels, and there is no definitive agreement regarding whether this biomarker is independent from LDL concentration [26]. Few studies have been performed in patients with type 2 diabetes and results are controversial. Nelson et al. recently reported that Lp-PLA2 predicts future risk of incident type 2 diabetes [27], but the Rotterdam Study did not find an association between the presence of diabetes and total Lp-PLA2 activity [28]. Regarding the association of Lp-PLA2 with cardiovascular disease, Hatoum et al. described that Lp-PLA2 activity was associated with incident coronary heart disease in patients with type 2 diabetes [29], but Nelson et al. reported that the Lp-PLA2 activity did not explain the excess of cardiovascular risk in diabetes [30]. To further complicate the matter, Kizer et al. found divergent associations between Lp-PLA2 activity and Lp-PLA2 mass and the risk of cardiovascular disease in a population with high prevalence of diabetes [31]. These authors suggest that a possible explanation for these paradoxical findings could be related to the distribution of Lp-PLA2 among lipoprotein classes. However, only three studies have previously analyzed the distribution of Lp-PLA2 between lipoproteins in diabetes. Kujiraoka et al. reported that, although no difference was observed in total Lp-PLA2 activity between patients with diabetes and control subjects, Lp-PLA2 distribution was altered in the former, with a lower proportion bound to HDL [32]. In a recent study, Mitsutake et al. obtained similar results in diabetic patients and also found that Lp-PLA2 in HDL was lower in subjects with increased coronary artery calcification [33]. Finally, Onat et al., which measured Lp-PLA2 mass, agreed with these authors and also found decreased content of this enzyme in HDL from diabetic patients [34]. However, none of these studies took into account the presence of phenotype B. Our data indicate that increased total Lp-PLA2 activity and abnormal distribution of Lp-PLA2 is strongly dependent on the presence of LDL subfraction phenotype B. The higher content of Lp-PLA2 in apoB-containing lipoproteins from patients with phenotype B is probably due to the higher affinity of this enzyme for the binding to sdLDL particles than to large, buoyant LDL particles [18]. The observation that Lp-PLA2 has a lower content in HDL from patients with phenotype B than from those with phenotype A concurs with the concept that this enzyme has an antioxidant role when is bound to HDL [35]. This could account for the increased concentration of oxLDL in diabetic patients with phenotype B.

## Conclusions

Our data agree with previous reports of alterations in the qualitative characteristics of LDL and HDL in patients with type 2 diabetes and demonstrate that some, but not all, of these abnormalities, are closely related to the presence of LDL subfraction phenotype B. Specifically, a high concentration of oxLDL and glLDL and a higher content of Lp-PLA2 in apoB-containing lipoproteins are favored by the presence of sdLDL particles. In addition, alterations observed in the composition of HDL from patients with phenotype B were stronger than in patients with phenotype A. The relevance of these results resides in the high prevalence of phenotype B among patients with poorly-controlled type 2 diabetes. Therapies focused on changing the LDL subfraction phenotype, such as fibrates [36] or glycemic optimization [37], would improve some qualitative pro-atherogenic characteristics of LDL and help to lower the increase in CVR present in type 2 diabetes.

## Competing interests

The authors declare that they have no competing interests.

## Authors’ contributions

J.L.S-Q. conceived of the study, wrote the manuscript and researched data. I.V. selected patients and reviewed the manuscript. E.J-F. researched data and reviewed the manuscript. J.S-H performed statistical analysis and reviewed the manuscript. R.B. researched data. F.B-V participated in the design of the study, contributed to the discussion and reviewed/edited the manuscript. J.O-L. participated in the design of the study, contributed to the discussion and reviewed/edited the manuscript. A.P. conceived of the study, selected patients and wrote the manuscript. All authors read and approved the final manuscript.
